# *In vitro* metabolic activation of vitamin D3 by using a multi-compartment microfluidic liver-kidney organ on chip platform

**DOI:** 10.1038/s41598-019-40851-9

**Published:** 2019-03-15

**Authors:** Jannick Theobald, Mohamed A. Abu el Maaty, Nico Kusterer, Bernhard Wetterauer, Michael Wink, Xinlai Cheng, Stefan Wölfl

**Affiliations:** Institute of Pharmacy and Molecular Biotechnology, Heidelberg University, Im Neuenheimer Feld 364, 69120 Heidelberg, Germany

## Abstract

Organ-on-chip platforms provide models that allow the representation of human physiological processes in cell-based miniaturized systems. Potential pre-clinical applications include drug testing and toxicity studies. Here we describe the use of a multi-compartment micro-fluidic chip to recapitulate hepatic vitamin D metabolism (vitamin D to 25-hydroxyvitamin D) and renal bio-activation (25-hydroxyvitamin D to 1,25-dihydroxyvitamin D) in humans. In contrast to cultivation in conventional tissue culture settings, on-chip cultivation of HepG2 and RPTEC cells in interconnected chambers, used to mimic the liver and kidneys, respectively, resulted in the enhanced expression of vitamin D metabolizing enzymes (CYP2R1, CYP27B1 and CYP24A1). Pump-driven flow of vitamin D3-containing medium through the microfluidic chip produced eluate containing vitamin D3 metabolites. LC-MSMS showed a strong accumulation of 25-hydroxyvitamin D. The chip eluate induced the expression of differentiation markers in HL-60 (acute myeloid leukemia) cells, assessed by qPCR and FACS analysis, in a manner similar to treatment with reference standards indicating the presence of fully activated 1,25 dihydroxyvitamin D, although the latter was not detected in the eluate by LC-MSMS. Interestingly, 25-hydroxyvitamin D by itself led to weak activation of HL-60 cells suggesting that 25-hydroxyvitamin D is also an active metabolite. Our experiments demonstrate that complex metabolic interactions can be reconstructed outside the human body using dedicated organ-on-chip platforms. We therefore propose that such systems may be used to mimic the *in vivo* metabolism of various micronutrients and xenobiotics.

## Introduction

Vitamin D deficiency is a global pandemic that affects approximately one billion people worldwide^1^. This phenomenon has broad implications since accumulating evidence has demonstrated that deficiency in this vitamin increases the predisposition to a myriad of chronic diseases, such as cardiovascular disease and cancer^1,2^.

The human body can produce 7-dehydrocholesterol (a provitamin), which in the skin is converted by UV light to previtamin D_3_ and further to vitamin D_3_. For the biosynthesis of its active form, vitamin D_3_ undergoes two hydroxylation steps, the first occurring in the liver, yielding 25-hydroxyvitamin D [25(OH)D], which is the most reliable biomarker for vitamin D status in humans^1^. The second hydroxylation step takes place in the kidneys, producing 1,25(OH)2D (also known as calcitriol), which then binds to the nuclear vitamin D receptor (VDR), eliciting major changes in gene expression patterns in target cells^1^. Cytochrome P450 (CYP) 2R1 (CYP2R1) has been previously shown to be a major, although not exclusive vitamin D-25-hydroxylase^3^, whereas CYP27B1 is known to be the 25(OH)D-1-hydroxylase^1^. Moreover, levels of both 25(OH)D and 1,25(OH)2D are feedback regulated, by the 25(OH)D-24-hydroxylase CYP24A1^4^.

Interest in this field has been sparked in recent years at least in part due to discovery of both VDRs and vitamin D-metabolizing enzymes in numerous cell types, highlighting a role for this molecule in non-classical target tissues^1^. In cancer cells for instance, calcitriol treatment regulates the expression of genes involved in cell cycle regulation, apoptotic signaling, differentiation, and nutrient metabolism^5–7^.

A major limitation in vitamin D-based chemotherapy is the need for supra-physiological doses to achieve substantial anti-tumor effects^6^. Such doses lead to hypercalcemia, hampering their clinical utility. To circumvent this obstacle, non-calcemic vitamin D analogs have been developed, and several have made the way to the clinic, such as maxacalcitol and paricalcitrol, which are both used to treat secondary hyperparathyroidism^8^. Furthermore, recent genome-wide association studies have identified single-nucleotide polymorphisms (SNPs) in genes encoding proteins involved in vitamin D synthesis, transport, and metabolism that influence vitamin D status in humans^9,10^. This is of major importance in determining subject-specific response to vitamin D supplementation, since different subjects possessing differing sets of SNPs would require varying doses of vitamin D compounds to elevate circulatory 25(OH)D levels to a desirable range, or to elicit specific biological effects. Therefore, reliable experimental systems are in demand to address these issues.

One category of *in vitro* models, which could address these issues is “organ-on-chip”, which are microfluidic platforms that aim to closely resemble different tissue types. By now, multiple organ-on-chip systems have been developed, reviewed in^11–15^.

Data have shown that culturing cells in microfluidic environments confers several advantages including enhanced cellular functionality. For example, hepatocytes cultured *ex vivo* quickly de-differentiate and lose their specific hepatic function^16,17^, whereas cells cultured in microfluidic platforms maintained the functionality of multiple CYP enzymes^18^. Additionally, CYP expression as well as albumin uptake of renal tubular epithelial cells in microfluidic setups are reported to be closer to their physiological counterparts^19,20^.

Early work, which include studies by *Koebe et al*. and *Ma et al*.^21,22^, has shown *in vitro* metabolism of drugs using mono-hepatocyte cultures. In case of the former, a hepatocyte bioreactor was combined with a microphysiometer system to assess extracellular acidification rate observed with hepatic metabolism. The latter study described a two-layered device where drug metabolite characterization and metabolism-induced cytotoxicity could be performed simultaneously. Studies utilizing microfluidic setups aiming to mimic tissue-tissue interactions include that of *Choucha*-*Snouber et al*., as well as our recent study^23,24^. In both studies, two-compartment devices were used to illustrate nephrotoxicity exhibited upon hepatic metabolism of a molecule.

We have also illustrated the potential use of microfluidic devices for continuous monitoring of CYP activity in living cells^25^ and the use of 3D multilayer platforms to mimic liver lobule^26^. In a more complex 3D based system, *Weber et al*. showed the recapitulation of proximal tubule microenvironment and functions, the secretory transport of organic solutes and the metabolism of 25(OH)D3^27^.

**Figure 1:**
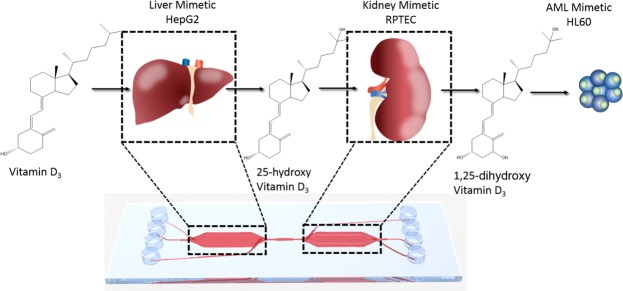
Schematic overview of vitamin D3 metabolism and bio-activation on-chip. Vitamin D3 is introduced into the microfluidic chip system and metabolised by the liver chamber giving rise to 25(OH)D3, which is then transported via the microfluidic flow to the kidney chamber where it is further metabolised to 1,25(OH)2D3. This bioactive form is then given to HL-60 cells and pro-differentiation effects of the molecule are investigated.

We therefore proposed that such multi-compartment microfluidic platforms may be adopted in vitamin D research for various applications, including investigating the effect/functionality of SNPs in the vitamin D pathway on the metabolism/bio-activation of the parent molecule and novel vitamin D prodrugs, as well as to study the influence of different dietary nutrients and xenobiotics on vitamin D metabolism. To investigate such hypothesis, we employed a custom two-compartment microfluidic chip, and seeded the first compartment with HepG2 cells, and the second with RPTEC cells, to mimic sequential hepatic and renal vitamin D metabolism, respectively. We postulated that this setup would enable cells to produce and release vitamin D metabolites, namely 25(OH)D and 1,25(OH)2D, into the eluate, which could then be applied on target cells to investigate the metabolites’ biological activities. In the current study, HL-60 (acute myeloid leukemia) cells were used to monitor the pro-differentiation effects of bioactive vitamin D, and thus provide evidence of the ability of the system to metabolize the vitamin (Fig. 1).

## Results

### Functionality of the two-compartment liver-kidney chip design and demonstration of fluid dynamics

**Figure 2:**
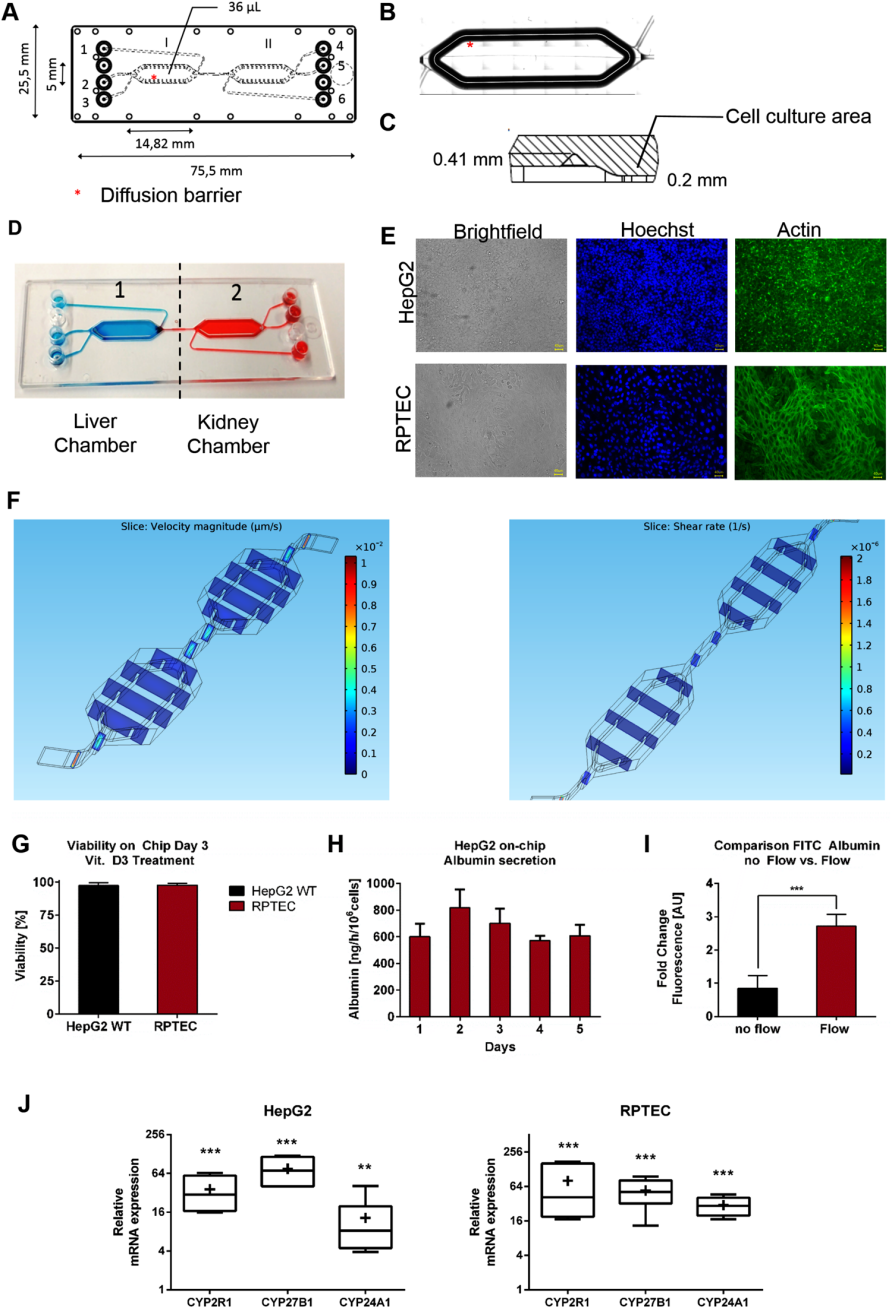
(**A**) Geometry of the two chamber microfluidic system. Chips are fabricated by microfluidic ChipShop Jena. (**B**) Position of the guidance barrier on the edge of the cell culture area. (**C**) Side view of the guidance barrier with specific height. (**D**) Illustration of the individual seeding of the two compartments of the Chip using food dye. (**E**) Brightfield picture and immufluorescent staining of HepG2 and RPTEC cells within the microfluidic system using Hoechst (blue) and Phalloidin Actin Dye (green). Images show confluent monolayer and sporadic 3D formations. (**F**) Simulation of flow velocity and shear rate in two chamber chip design using flow rates of 20 µL/h. Shear rate and velocity flow rate were highest in the narrow inlet and outlet part of the chambers. Shear rate within the culture are was calculated to be as low as 0.05 Pa. (**G**) Viability of HepG2 and RPTEC cells on-chip after 3 days of culture. Values are calculated as ratio of PI-negative cells divided by total cell number. Numbers are calculated in percentage as average of n = 3 Chips with 20 Pictures per Chip per chamber. (**H**) Albumin secretion of HepG2 cells cultured in microfluidic chips. Albumin secretion remained constant over the culture period (5 days), with a concentration of >600 ng/h/10^6^ cells. Data presented are the average of 3 independent experiments. Error bars ± SD. (**I**) Comparison of FITC-Albumin uptake of RPTEC cells under static and fluidic conditions. Values are plotted as fold change in fluorescence intensity [AU]. Analysis of fluorescence intensity was performed using ImageJ. (**J**) Comparison of CYP mRNA expression under static and microfluidic conditions for HepG2 and RPTEC cells using RT-qPCR. Relative expression values are calculated using the ∆∆Ct method with RPL-30 as the reference gene. Lines in the middle of the box plot represent the median, whereas the + sign represents the mean of more than 3 independent experiments. Error bars ± SD. Statistical significance between the different culture conditions was calculated using an unpaired two-tailed Student’s t-test, where p-values less than or equal to 0.05, 0.01, and 0.001, depicted as *, **, and ***, respectively.

A two-chamber microfluidic platform was used. The individual chambers of the two-compartment microfluidic platform exhibited a length of 14,28 mm, a width of 5 mm, and a total volume of 36 µL. The two compartments were connected by a 150 µm narrow channel (Fig. 2A). The cultivation areas in the chambers are surrounded by guidance barriers which shield the cell culture area from the high shear rate of the fluidic flow and in addition serve as an air bubble trap (Fig. 2B,C)^23^. Using our previously reported seeding protocol^23^, each chamber could be seeded individually, which allowed distinct separation of the liver and kidney cells in the individual compartments (Fig. 2D). Cell culture areas were coated with 0.4% Collagen solution to ensure ECM formation as previously reported^17,28^.

Microscopical investigation of the cells in the compartments revealed confluent monolayers with sporadic multilayer formation (Fig. 2E). After three days in dynamic culture, cellular viability was as high as 97.2% ± 2.2% and 97.8 ± 1.3% for HepG2 and RPTEC cells, respectively (Fig. 2G).

The fluid dynamics of the chip were analyzed using the flow simulation software COMSOL Multiphysics, as previously reported^26^. The applied flow rate of 20 µL/h matched the simulated one. This low flow rate was used to facilitate the metabolic process, since our system was employed mono-directionally with no re-circulation. Flow velocity and shear rate were found to be highest in the narrow inlet/outlet as well as in the connection between the chambers. Within the cell culture area, low shear rate (0.05 Pa) and velocity was detected (Fig. 2F).

We then evaluated the functionality of liver cells by measuring albumin production, and observed a constant albumin secretion during the culture period, declining levels as previously reported for static systems^23,26^ were not detected (Fig. 2H). Uptake of FITC-Albumin was investigated as a marker for kidney cell activity^23^. Significant increase in the uptake was found under fluidic flow, compared to static culture (Fig. 2I). Additionally, we investigated the effect of on-chip cultivation on the mRNA expression levels of vitamin D-metabolizing CYPs (CYP2R1, CYP27B1, CYP24A1) in RPTEC and HepG2 cells, and compared them to that of cells maintained in static culture conditions. Indeed, expression levels of these enzymes were found to be significantly induced with on-chip cultivation (Fig. 2J). This is in line with our previous observation that the basal mRNA expression levels of various CYPs are induced with on-chip cultivation^23^. We further investigated the epithelial behavior of RPTEC cells by comparing the mRNA expression of Occludin, E-Cadherin and ZO-1 for cells cultured under static and under microfluidic conditions and observed enhanced expression of these markers with on-chip cultivation (Supplementary Fig. 1). Altogether, these results clearly highlight the advantages the microfluidic setup imparts on cellular functions.

### The eluate of vitamin D3-supplied liver-kidney chips induces differentiation of HL60 cells more potently than the parent molecule

**Figure 3:**
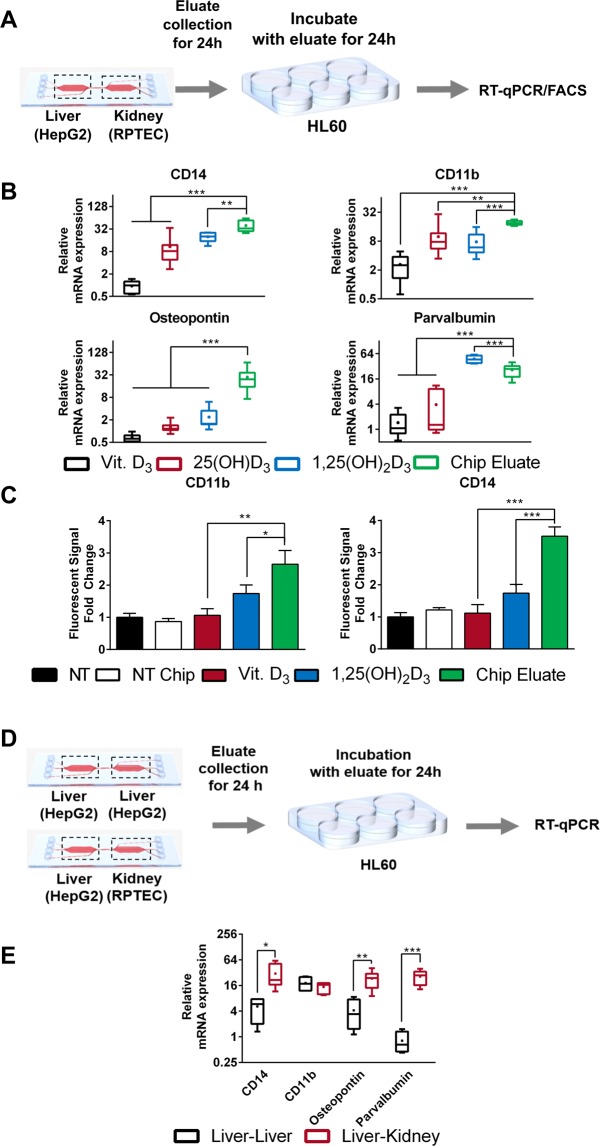
(**A**) Explanatory workflow using the eluate of the chip system to treat HL60 cells for 24 hours and perform RT-qPCR analysis shown in (**B**) and FACS shown in (**C**). (**B**) Effect of various treatments on the mRNA expression of multiple differentiation markers (CD14, CD11b, Osteopontin and Parvalbumin) in HL60 cells using RT-qPCR. Note that chip eluate refers to the eluate of a chip containing both HepG2 and RPTEC cells in subsequent chambers, which was fed with medium containing vitamin D3. (**C**) Analysis of CD11b and CD14 protein levels in HL-60 cells in response to different treatments using FACS. Values are presented as fold change of fluorescence intensity normalized to fluorescence intensity non-treated cells. Values are the mean of two biological replicates. Error bars ± SD. (**D**) Explanatory workflow to investigate the effect of the kidney chamber in the two compartment microfluidic setup. (**E**) Comparison of the expression of HL-60 differentiation markers, treated with the eluate of a HepG2-liver-chip only (black) and a HepG2-RPTEC liver-kidney chip (red). For all figures lines in the middle of the box plot represent the median, whereas the + sign represents the mean of more than 5 independent experiments Error bars ± SD Statistical significance between the cells treated with the chip eluate and other treatments was calculated using an unpaired two-tailed Student’s t-test, where p-values less than or equal to 0.05, 0.01, and 0.001, depicted as *, **, and ***, respectively.

Among the known anti-tumor effects mediated by 1,25(OH)2D3 is its ability to induce differentiation in multiple tumor types, such as prostate, breast, and myeloid leukemia cells^2^. To investigate the possibility that the liver-kidney microfluidic chip metabolizes vitamin D3 into its bioactive form, we continuously introduced medium containing either vitamin D3 (20 µM final concentration) or DMSO at a flow rate of 20 µL/h, for a period of 24 h (Fig. 3A). After passage through the chip the eluted medium was collected and used to treat HL60 cells for 24 h. Total RNA was then isolated from cells, and mRNA expression levels of various differentiation markers, including CD11b, CD14, osteopontin, and parvalbumin, were assessed using RT-qPCR. In parallel, to provide suitable controls for comparisons, HL60 cells were treated with the different vitamin D3 metabolites— 25(OH)D3 (2 µM), and 1,25(OH)2D3 (100 nM)—as well as vitamin D3, under standard treatment conditions.

HL60 cells treated with the eluate of vitamin D3-treated chips exhibited enhanced mRNA expression of all investigated differentiation markers, compared to cells treated with medium eluted from DMSO-treated chips. Moreover, cells treated with the eluted medium of vitamin D3-treated chips exhibited higher expression levels for all investigated genes in comparison to reference treatment with vitamin D3, indicating metabolic transformation. Further comparison to treatments of HL-60 cells with either 25(OH)D3 or 1,25(OH)2D3 under standard conditions, revealed a mostly higher induction of differentiation markers in cells treated with the medium eluate of the vitamin D3-feeded liver-kidney chip, indicating the presence of one or more vitamin D metabolites in the eluate of treated chips (Fig. 3B).

To verify these observations, we also analyzed the presence of differentiation markers on the protein level, using the cell surface markers CD11b and CD14. HL60 cells cultivated in DMSO-supplied chip eluate did not exhibit statistically significant differences in protein expression of these differentiation markers compared to cells cultivated in standard DMSO-treated medium. However, treatment with vitamin D3-containing chip eluate led to significantly higher protein expression of CD11b and CD14 in comparison to DMSO-treated cells, DMSO-treated chip eluate, as well as compared to standard treatments with either vitamin D3 or 1,25(OH)2D3 (Fig. 3C).

Based on observations obtained in previous studies^23,29^, we postulated that on-chip cultivation may have led to an increased half-life of vitamin D3 metabolites due to the continuous production and secretion of albumin by HepG2 cells (Fig. 2H), which may have led to the more sustained induction in differentiation markers compared to standard treatments performed in serum free medium. To investigate this hypothesis, we treated HL60 cells with 1,25(OH)2D3 (100 nM) and different concentrations of bovine serum albumin (BSA; including the concentration of albumin found in the HepG2 chip eluate Fig. 2H). We did not observe significant differences in the regulation of expression of the different target genes by 1,25(OH)2D3 with different BSA concentrations (Supplementary Fig. 2). Therefore, the enhanced induction in the expression of these genes with the chip eluate may be due to other factors, such as the presence of different vitamin D metabolites in the eluate, or the secretion of factors by the liver and/or kidney chambers in response to vitamin D3 in the medium that may have led to the potentiated induction in expression.

To investigate the necessity of both the liver and kidney cells in metabolizing vitamin D3, we seeded the two chip compartments with only HepG2 cells (liver-liver) and with HepG2-RPTEC (liver-kidney) (Fig. 3D), and performed DMSO and vitamin D3 treatments as described earlier, after which the eluate was collected and used to treat HL-60 cells. The mRNA expression levels of differentiation markers, except CD11b, were more potently induced by the liver-kidney chip (Fig. 3E), illustrating the distinctive effect of consecutive metabolism.

We further investigated on the effect of the close proximity of the liver-kidney system and the effect of the microfluidic flow and compared the induction of the differentiation markers for cells treated with static supernatant of HepG2 cells, static supernatant of HepG2-RPTEC cells and the chip eluate. For the static condition, medium was aspirated after 24 hours and put on HL60 cells for the HepG2 and on RPTEC cells for additional 24 hours for the HepG2. The results indicate that for all four markers, induction was highest using the chip eluate, in case of CD11b slight induction was also observed using static treatments (see Supplementary Fig. 3).

### Treatment with the chip eluate regulates CYP expression in target tissues

Since we observed a clear induction in the expression of vitamin D metabolizing CYPs with on-chip cultivation, we postulated that on-chip treatment may also lead to altered CYP regulation compared to standard treatments, which could explain the differential regulation of differentiation markers observed.

**Figure 4:**
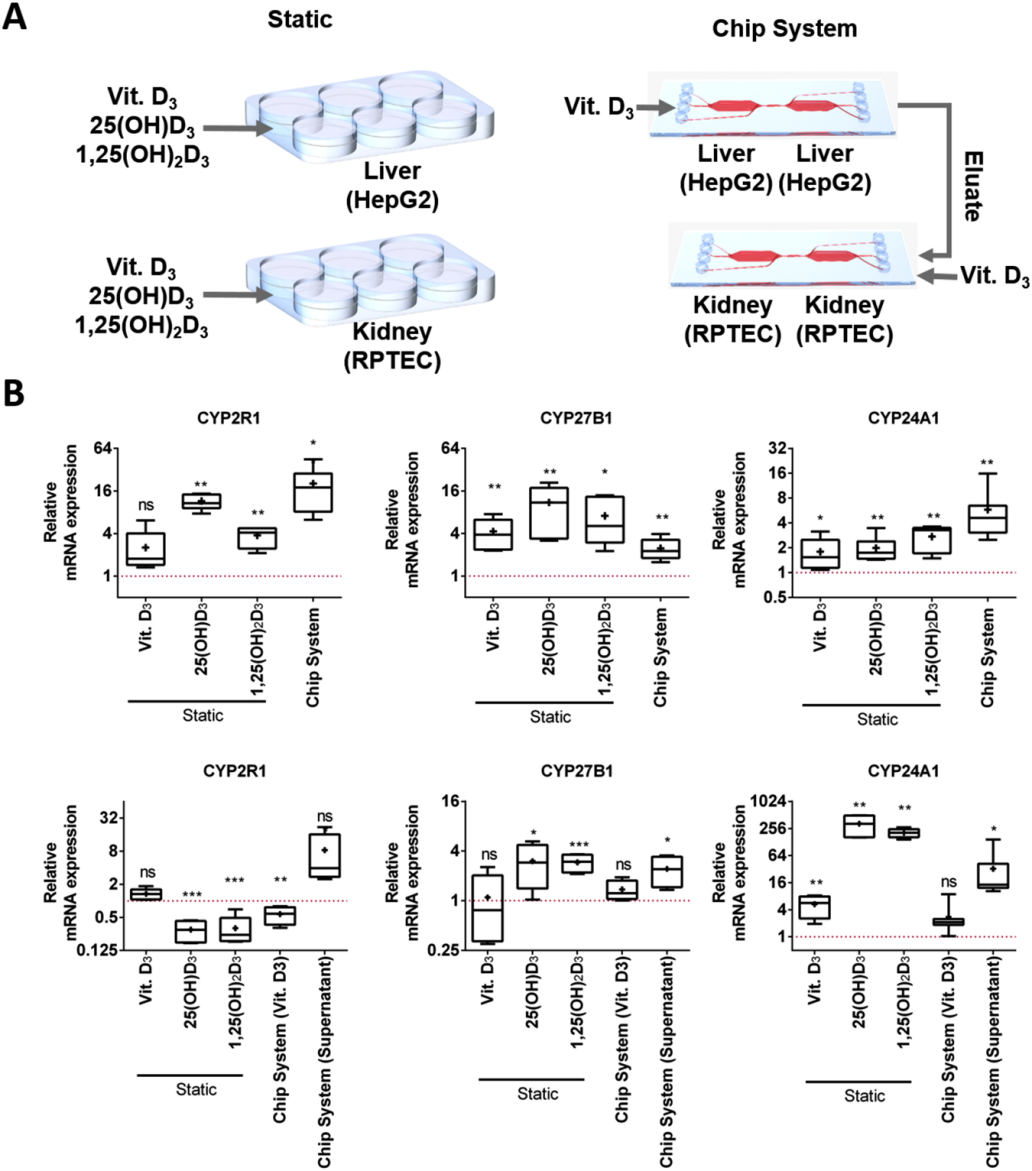
(**A**) Illustration of the performed experiments investigating regulation of various CYPs by treatment of HepG2 and RPTEC cells cultured in static and microfluidic setups. (**B**) Comparison of CYP mRNA expression in HepG2 (upper row) and RPTEC cells (lower row). In the upper row Chip System refers to a HepG2 chip treated with vitamin D3, in the lower row Chip System (Vit. D3) refers to a RPTEC chip treated with vitamin D3 and Chip System (Supernatant) refers to a RPTEC chip treated with the eluate of a HepG2 chip fed with vitamin D3. The different treatments are illustrated in A. Values for HepG2 and RPTEC cells were obtained using RT-qPCR. Relative expression values are calculated using the ∆∆Ct method with RPL-30 as the reference gene. Lines in the middle of the box plot represent the median, whereas the + sign represents the mean of more than 5 independent experiments. Error bars ± SD. Statistical significance between the different culture conditions was calculated using an unpaired two-tailed Student’s t-test, where p-values less than or equal to 0.05, 0.01, and 0.001, depicted as *, **, and ***, respectively.

Therefore, we investigated the changes in mRNA expression patterns of the various CYPs involved in vitamin D3 metabolism in response to on-chip treatment, and compared them with those observed in cells treated under standard cell culture conditions. Medium containing either DMSO or vitamin D3 (final concentration 20 µM) was continuously introduced (flow rate: 20 µL/h) to chips only containing HepG2 cells for a period of 24 hours (reaching a final volume of 480 µL). A similar setup was repeated with RPTEC-seeded chips, however an additional treatment condition was included, in which chips were fed culture medium eluted from chips containing either DMSO- or vitamin D3-treated HepG2 cells (Fig. 4A). Total RNA was then extracted from chips and changes in mRNA expression of the different CYPs were investigated using RT-qPCR. Furthermore, the same cell lines were treated for 24 hours with vitamin D3 (20 µM) and its metabolites—25(OH)D3 (2 µM), and 1,25(OH)2D3 (100 nM)—in static conditions, and changes in CYP mRNA expression levels were investigated (Fig. 4B).

Similar and disparate effects on the mRNA expression of the investigated CYPs between HepG2 cells treated in static conditions with various vitamin D3 molecules and on-chip treated cells were observed. With regards to CYP24 A1 regulation, all treatments independent of culture method were found to induce mRNA levels. However, on-chip treated HepG2 cells exhibited comparable CYP24A1 mRNA expression to cells treated with either vitamin D3 metabolite in static conditions, but significantly higher levels compared to those treated with vitamin D3. Similarly, CYP2R1 induction in HepG2 cells treated on-chip was found to be more significant than that observed in cells treated with either the parent or the active molecule, however in line with that observed with 25(OH)D3 treatment. Regulation of CYP27B1 on the other hand was found to be similar in cells independent of culture and treatment conditions (Fig. 4B upper row).

RPTEC cells treated with vitamin D3-containing medium eluted from HepG2 cells were found to express significantly different CYP24A1 and CYP2R1 mRNA levels compared to cells treated with either vitamin D3 metabolite in static conditions. Interestingly, looking at the same genes in RPTEC cells, on chip culture treated directly with vitamin D3 exhibited significantly different mRNA levels compared to chip cultures treated with vitamin D3-containing HepG2 eluate, indicating the presence of metabolic products in the HepG2 eluate. With regards to CYP27B1 mRNA levels, statistically insignificant differences were observed between chips treated with vitamin D3-containing eluent and those treated with vitamin D3, as well as compared to cells exposed to the various treatments in static conditions (Fig. 4B).

### Detection of vitamin D3 metabolite(s) in the liver-kidney chip eluate

In view of these data, we aimed to investigate whether vitamin D3 metabolites were produced and secreted by cells in the microfluidic chip. To this end, we performed LC-MSMS to assess the presence of either vitamin D3, 25(OH)D3, 1(OH)D3, or 1,25(OH)2D3 in the eluted medium of treated chips. A previously described method was used^30^, with minor modifications, to achieve base-line separation of all analytes of interest, after which the fragmentation patterns of the standards were determined. Noteworthy is that both the parent molecule and the bioactive form exhibit short half-lives, in contrast to 25(OH)D^31^, which is also reflected in the use of blood plasma levels of this metabolite to determine the vitamin D status in humans.

**Figure 5:**
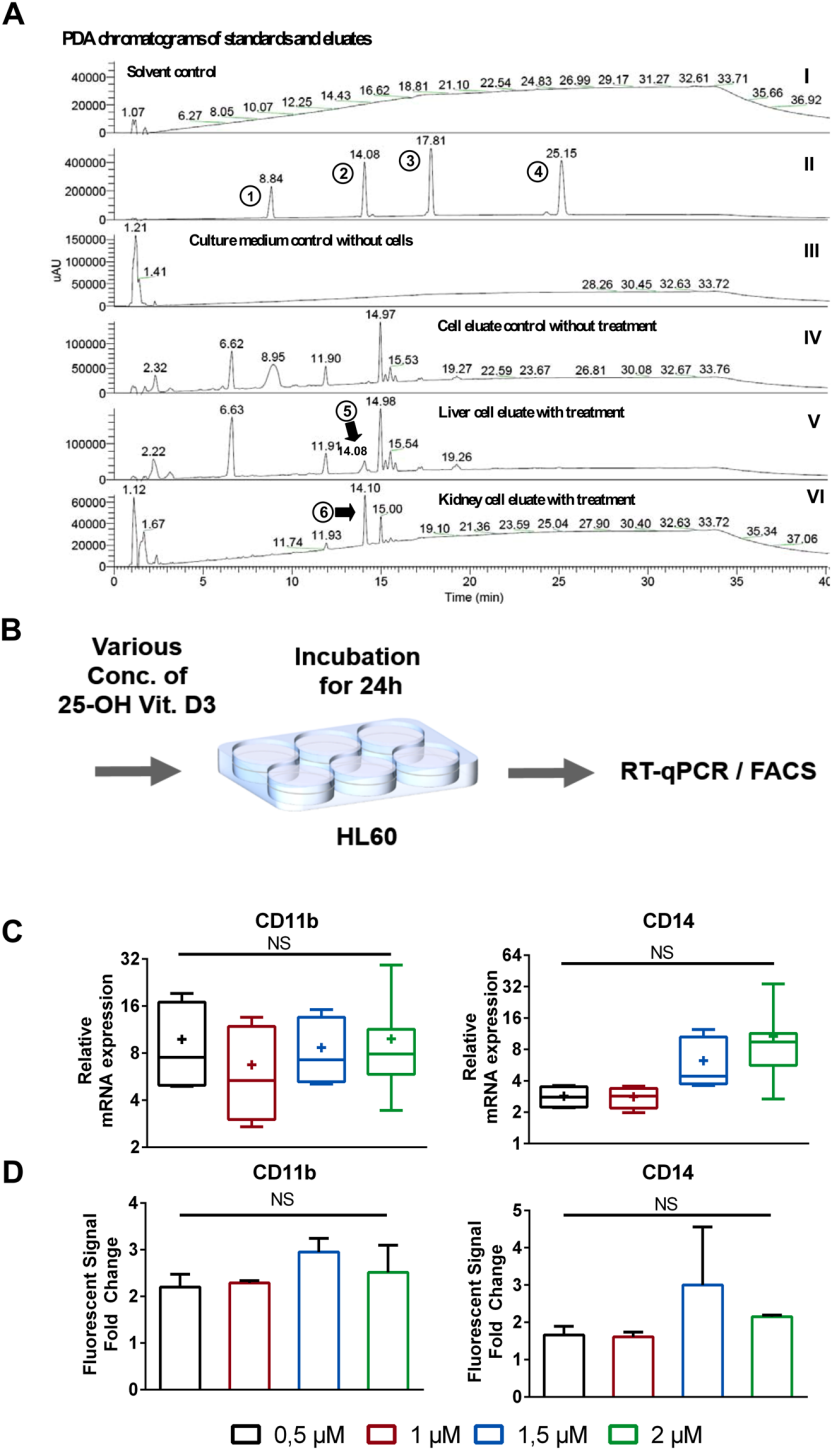
Verification of biosynthesis of 25(OH)D_3_ after addition of 20 µM vitamin D3 in microfluidic eluate by LC-MSMS. (**A**) PDA chromatograms; I: MeOH negative control; II: Standards 1,25(OH)D_3_ (1), 25(OH)D_3_ (2), 1(OH)D_3_ (3), and vitamin D_3_ (4); III: Cell culture medium pure; IV: Culture medium of cell culture without treatment; V: Eluate of HepG2 microfluidic chip fed with cell culture medium containing 20 µM vitamin D3; VI: Eluate of RPTEC microfluidic chip fed with cell culture medium containing 20 µM vitamin D3. (Peak analysis in supplementary Fig. 4). (**B**) Explanatory workflow to determine the influence of 25(OH)D3 concentration on HL60 cells for using RT-qPCR shown in (**C**) and FACS shown in (**D**). (**C**) Effect of various concentration of 25(OH)D3 on the mRNA expression of CD14, CD11b in HL60 cells using RT-qPCR. (**D**) Analysis of CD11b and CD14 protein levels in HL-60 cells in response to different concentration of 25(OH)D3 using FACS.

For the analytical determination of metabolites the same treatment protocol as described in the previous section (illustrated in Fig. 4A) was used, and vitamin D metabolites were extracted from the eluted medium. A peak corresponding to 25(OH)D3 was detected in the medium eluted from both the liver-liver and the liver-liver-kidney-kidney chips (Fig. 5A) Neither vitamin D3 nor 1,25(OH)2D3 were detected in the chip eluates, possibly due to either their short half-life or technical limitations (e.g. levels below limit of detection of the chromatographic method, or low extraction yield) (Fig. 5A).

The obtained MS spectra of the peaks were compared to the ones of standard substances, and an accurate match of masses was observed, confirming the metabolism of vitamin D3 to 25(OH)D_3_ (Supplementary Fig. 4) Based on the comparison with the standard calibration curve, a lower concentration of 25(OH)D3 was found in the eluate of the liver-liver-kidney-kidney chip compared to the concentration in the eluate of the liver-liver chip (1.26 µM vs 1.48 µM 25(OH)D3) (Supplementary Fig. 4).

We then decided to revisit the discrepancy in the induction of the differentiation markers when comparing the chip eluate and reference vitamin D3 metabolites (Fig. 3B,C). HL60 cells were treated with various concentrations of 25(OH)D3 and the mRNA and protein expression levels of CD14 and CD11b were analyzed using RT-qPCR and FACS, respectively (Fig. 5B). To our surprise even though 25(OH)D3 clearly induces the expression of the differentiation markers, the tested concentrations had no major effects on the expression level in our experiments (Fig. 5C,D). Although only 25(OH)D3 could be detected analytically, the biological effects of the eluate clearly demonstrate the capacity of our system to metabolize and secrete activated vitamin D3.

## Discussion

A plethora of *in vitro* and preclinical studies have demonstrated vitamin D effects beyond bone mineralization. In oncology, data illustrate the ability of this molecule to induce apoptosis and differentiation, modulate metabolism, as well as inhibit proliferation and metastasis^2,5–7,32^. However, several issues complicate the translation of these findings to the clinic, including the hypercalcemia associated with high calcitriol doses^8^, as well as the presence of SNPs in the vitamin D pathway that influence vitamin D status and possibly response to supplementation^9,10^. Moreover, the inconclusiveness of recent vitamin D randomized-controlled trials evaluating the molecule’s anti-cancer potential has been attributed to the lack of increase in serum 25(OH)D levels in response to supplementation^33^, which may be due to different vitamin D metabolizing efficiencies of the recruited subjects. We propose that the experimental setup described here enables comprehensive tackling of these issues, since cell lines genetically modified to harbor common variants in vitamin D metabolizing genes may be used to investigate the functionality/effect of such mutations on vitamin D metabolism, as well as that of novel vitamin D pro-drugs. Additionally, the system could be used to study the impact of different drugs and nutrients on vitamin D metabolism, which may influence the outcomes of supplementation.

Here we demonstrate the use of our two-compartment microfluidic platform for mimicking vitamin D3 metabolism *in vitro*. We show that passing vitamin D3 through two consecutive chambers, resembling the liver and kidneys, yields metabolic products that induce anti-cancer effects in acute myeloid leukemia cells, in a manner similar to that observed with the bio-active form of vitamin D3. Finally, we analyzed the differentiation of acute myeloid leukemia cells (HL60 cells), as a model for vitamin D3 anti-cancer activity.

Indeed, medium eluted from chips initially treated with vitamin D3 was found to induce the expression of differentiation markers in HL-60 cells in a manner more potent than that observed with standard vitamin D3 treatment. The changes observed were similar to that obtained by calcitriol treatment, indicating the possible occurrence of complete metabolic activation. Additionally, comparing the mRNA expression of relevant CYPs between cells seeded on-chip versus standard cell culture dishes highlights the improved metabolic profile in the micro-fluidic setting. Moreover, we detected metabolic products of vitamin D3 using HPLC/MS. Altogether, our data signify that the described microfluidic chip can be used to mimic vitamin D3 metabolism *in vitro*.

First we illustrate the efficient and complete metabolism of vitamin D3 by cells in the microfluidic device, and demonstrate differential cellular responses between on-chip and static treatments (Fig. 3B). We postulate that these discrepancies may be explained by a number of factors observed in this study as well as previously reported results by others^23,26,34^, including enhanced albumin secretion and uptake (see Fig. 2H and I), as well as altered CYP expression in response to shear stress (Fig. 2J). Additionally, gradual introduction of culture medium containing vitamin D3 to cells in the microfluidic device, as opposed to single high volume treatments performed on cells cultured in static conditions, may have led to altered regulation of particular CYPs. For example, on-chip vitamin D3 treatments led to higher CYP24A1 mRNA levels in HepG2 cells in comparison with the same treatment in static conditions.

Similarly, the altered regulation of CD11b and CD14 expression in HL-60 cells treated with vitamin D3-containing chip eluent compared to standard treatments was initially postulated to be the result of an extended half-life of the metabolites in the eluted medium, which was found to contain higher albumin levels compared to medium supernatant of static conditions. In such scenario, metabolic products released from chips would be less “free” and more “bound” to albumin—and vitamin D binding protein—enabling a slower, more sustained activation of differentiation markers in HL-60 cells compared to the rapid and potentially transient regulation seen with treatments performed in serum-free medium. However, we observed that increasing concentrations of BSA in the culture medium do no impact 1,25(OH)2D3’s regulation of its target genes. Therefore, the enhanced induction in the expression of the investigated genes with the eluate treatment may be due to the secretion of factors by cells in the microfluidic chip that potentiate vitamin D’s regulation of its target genes.

In conclusion, we propose that the experimental setup described in this report could be exploited in studies investigating the metabolism and activities of vitamin D, as well as other micronutrients and xenobiotics.

## Materials and Methods

### Cell culture

HepG2 cells, RPTEC cells and HL60 cells were obtained from ATCC (ATCC, Manassas, USA). Before use in the microfluidic system cells were maintained following standard mammalian tissue culture protocols. In short, cells were maintained in T25 flasks (Corning, Kaiserslautern, Germany) in DMEM high glucose containing 10% fetal bovine serum and 1% Penicillin-Streptomycin (10.000 U/ml) (Gibco Thermo Fisher Scientific, Germany) in a conventional humidified tissue culture incubator at 37 °C and 5% CO_2_. As reported before^23^, cells were maintained at low passage number <7 and cells were split at confluencies of >85% using standard cell culture procedures.

### Microfluidic system

A detailed characterization of the Chip design, the seeding pattern, the fluid dynamics and the applied fluidic propulsion system had been described previously^23^. For clarity a description of the main components of the system and parameters for cell seeding and cultivation are also provided here. The system is based on a two-chamber interconnected microfluidic chip, fabricated by ChipShop^23^. For microfluidic flow chips are connected with low pressure nEMESYS syringe pumps (Cetoni, Germany).

### Cell seeding in microfluidic flow chips

Following the same procedure as previously described^23^, before seeding chambers of the microfluidic chips were coated with collagen for one hour at 37 °C using a 1:3 dilution of a 0.4% Collagen R solution (Serva, Germany) in PBS. The solution was then washed out and thoroughly removed using PBS. Subsequently HepG2 cells and RPTEC cells were seeded in the respective chambers at 6 × 10^5^ cells, resulting in dense cell layer in each compartment (chamber). To ensure that cells did not mix between the chambers of the chip, an optimized seeding pattern was used^23^, After seeding, the two-chamber chips were placed in an incubator for 4 h. Then medium was replaced and cells seeded in the chips were further incubated overnight in static conditions (without flow) at 37 °C in humidified atmosphere, 5% CO_2_ to ensure formation of a stable cell layer before introduction of medium flow^23^.

### Culture conditions in microfluidic flow chips

Following the overnight incubation in static conditions microfluidic flow was applied using a constant microfluidic flow of 20 µL/h. For this, the low pressure nEMESYS syringe pump (Cetoni Germany) was set up in a combination with a 5.0 mL syringe and a H 1/4″-28 Tubing Connector (ILS, Germany). For coupling to the microfluidic chip PEEK tubing (inner diameter 1/32″) was used, and connected to chips with a miniLuer adapter (ChipShop, Jena) as described^23^. The unidirectional medium flow was applied via the HepG2 chamber providing a “liver to kidney” flow direction and the flow through (supernatant) was taken after medium also passed through the RPTEC chamber.

### Microfluidic cell culture for metabolism studies

For metabolism analysis under fluidic conditions, HepG2 cells and RPTEC cells were cultured in microfluidic chip chambers at 37 °C with 5% CO_2_ and supplied with DMEM high glucose without phenol red, and without adding fetal bovine serum or Penicillin-Streptomycin.

### HL60 treatment preparations

For the analysis of the HL60 differentiation, HL60 were also cultured in DMEM high glucose without phenol red, and without fetal bovine serum for 48 hours. After cells were treated with flow through (supernatant) from the liver-kidney chip, pure control medium or medium supplemented with controls. After 24 h incubation HL60 cells were analyzed using RT-qPCR or flow cytometry.

### RT-qPCR

RNA was extracted using the trizol RNA extraction protocol as described^5^. Quantitative reverse transcription real-time-PCR (RT-qPCR) was performed using standard protocols in accordance to manufacturer’s recommendations on Lightcycler 96 (Roche, Germany). After RNA was isolated from cells on chips or normal cell culture, cDNA was generated by reverse-transcription of equivalent amounts of total RNA using a cDNA synthesis kit (ProtoScript®, NEB, Germany) and RT-qPCR was carried out using a PCR master mix with SYBR Green fluoresce (qPCRBIO SyGreen Mix Lo-Rox, Nippon Genetics, Germany) as described^23^. PCR-primers were obtained from Eurofins (Germany). The primer sequences used are listed in supplementary information. Actin and RPL30 were used as endogenous reference control.

### Flow cytometry

FACS was performed using a Guava easyCyte Flow Cytometer (Merck, Germany) and round bottom 96 well plates (Corning, USA). HL60 cells incubated with various treatments for 24 h were washed with PBS and fixed with 4% PFA for 1 h. Primary antibody against CD11b, CD14 (SantaCruz Biotechnology, Germany) was diluted 1:500 and added to the cells and incubated at 4 °C over night. Cells were washed 3 times by centrifugation at 200 g for 5 min and washed with PBS. Fluorescent-labeled secondary antibody was diluted 1:2000, cells were re-suspended in the solution and incubated for 1 h at room temperature in the dark. Cells were again washed by centrifugation and PBS for 3 times and diluted to a concentration of 1 × 10^6^ cells/ml.

### Detection of albumin with ELISA

Secretion of albumin by HepG2 cells was detected using an ELISA kit (Bethyl laboratories, Montgomery, TX). The assay was performed according to the manufactures protocol. As earlier described^23,26^, the flow through of the microfluidic chips was collected on daily basis and stored at −20 °C prior to performing the assays. The protein content of samples was determined using Bradford assay (Sigma Aldrich, Germany), before samples were diluted in the supplied buffer. 96-Well flat bottom plates were used to perform the assay. Absorbance at λ450nm was measured using a microplate reader (Ultra TECAN plate reader, Germany)^23^.

### Analysis of albumin uptake

The tubular kidney cells uptake properties of RPTEC cells were tested with an albumin-fluorescein isothiocynate conjugate (FITC-Albumin) under static and fluidic conditions as described^23^, adapting the protocol of Ferrell *et al*.^20^. In brief both fluidic and static culture were albumin starved for 24 h prior to treatment. Cells were exposed to FITC-albumin containing medium for 3 hours. Subsequently cells were thoroughly washed with PBS and fluorescence pictures were taken using a Keyence BZ 9000 fluorescence microscope. Fluorescence intensity was analysed using ImageJ software^23^.

### LC-MSMS analysis

Sample preparation was started by protein precipitation with ethanol (pH 2.2), followed by centrifugation at 12.000 × g for 30 min. Next, a two phase separation with ethyl acetate was used, the ethyl acetate vaporized using a Rotavap (Büchi, Germany), and the residues reconstituted in methanol. LC-MSMS analysis was performed on a Finnigan LCQ-Duo ion trap mass spectrometer with an ESI source (ThermoQuest) coupled to a Finnigan Surveyor HPLC system (MS pump plus, auto sampler, and PDA detector plus) with a EC 150/3 Nucleodur 100–3 C18ec column (Macherey-Nagel). A gradient of water and acetonitrile (ACN) with 0.1% formic acid each was applied from 50% to 100% ACN in 15 min, isocratic with 100% ACN for 15 min, and return to 50% ACN in 15 min again. The method was driven with a column temperature of 40 °C, a flow rate of 0.5 ml/min, and the injection volume was 20 µl. All samples were measured in the positive (ESI+) mode. The MS was operated with a capillary voltage of 10 V, source temperature of 240 °C, and high purity nitrogen as a sheath and auxiliary gas at a flow rate of 80 and 40 (arbitrary units), respectively. The ions were detected in a mass range of 50–2000 m/z. Collision energy of 35% was used in MS/MS for fragmentation. Data acquisitions and analyses were executed by Xcalibur^TM^ 2.0.7 software (Thermo Scientific). For quantification, the 25(OH)D3 peak in the eluate was compared to the standard calibration curve (linear range: 1–312 µM; R² > 0.995). To maintain the accuracy of retrofit quantification eluate samples and standards were run in parallel.

## Supplementary information

Supplementary information
