# Supplementary material for: In vitro metabolic activation of vitamin D3 by using a multi-compartment microfluidic liver-kidney organ on chip platform
Source: Sci Rep. 2019 Mar 15;9:4616. doi: 10.1038/s41598-019-40851-9 (PMC6420623; doi:10.1038/s41598-019-40851-9)
Supplement: Supplementary file 1 — Supplementary information [file 41598_2019_40851_MOESM1_ESM.pdf]

In vitro metabolic activation of vitamin D3 by using a multi-compartment microfluidic liver-kidney organ on chip platform

Jannick Theobald, Mohamed A. Abu el Maaty, Nico Kusterer, Bernhard Wetterauer, Michael Wink, Xinlai Cheng and Stefan Wölfl*

Institute of Pharmacy and Molecular Biotechnology, Heidelberg University, Im Neuenheimer Feld 364, 69120, Heidelberg, Germany

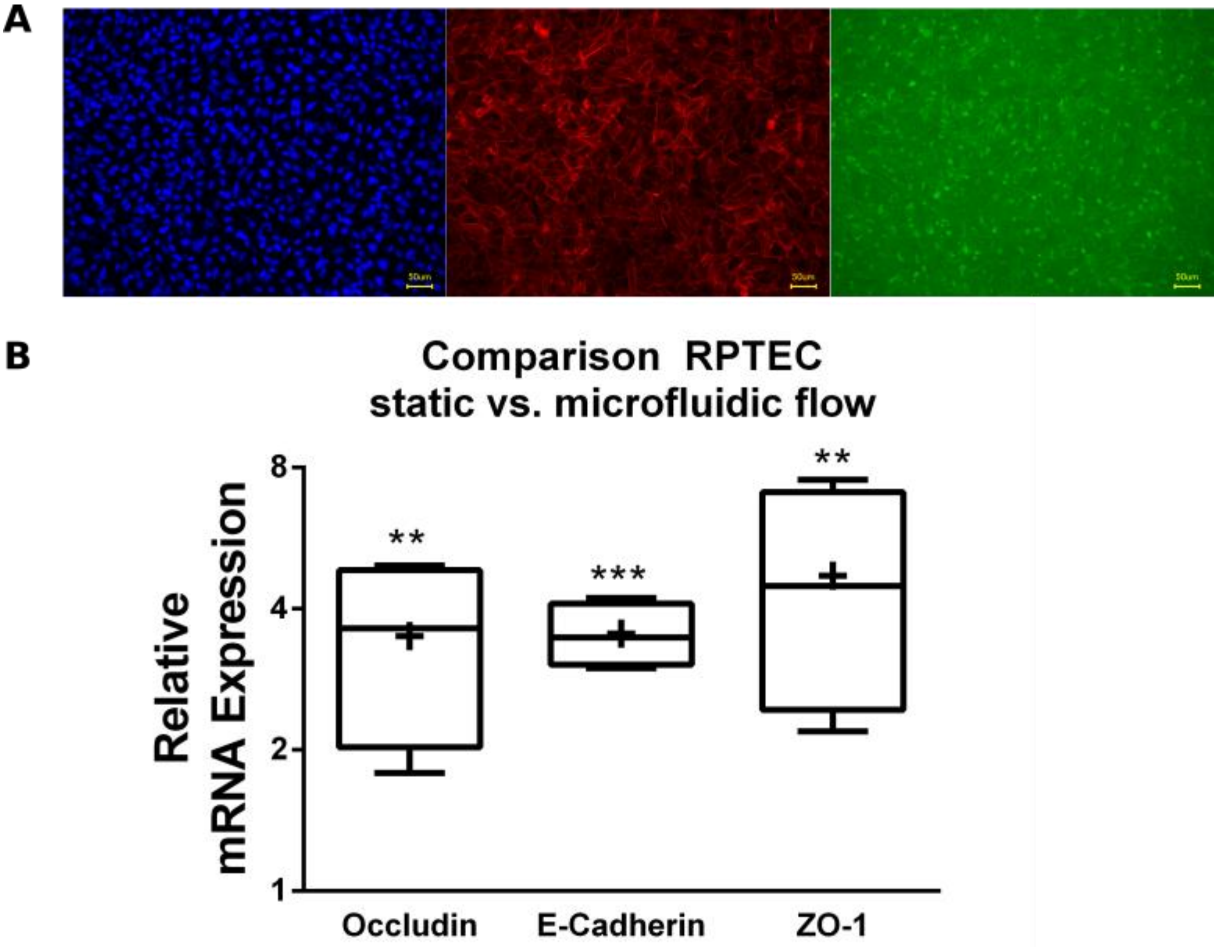

Supplementary Figure 1 ***A*** *Immunostaining of RPTEC cells in the chip using Hoechst (blue), Actin (red) and E-cadherin (green) staining. Cells were cultured for 72 hours in the chip.* ***B*** *Comparison of the expression of different epithelial cell marker in RPTEC cells. Expression was compared between cells cultured under microfluidic conditions for 48 hours and cells cultured under static conditions. For all figures lines in the middle of the box plot represent the median, whereas the + sign represents the mean of more than 4 independent experiments. Statistical significance between the cells treated with the chip eluate and other treatments was calculated using an unpaired two-tailed Student's t-test, where p-values less than or equal to 0.05, 0.01, and 0.001, depicted as *, **, and ***, respectively.*

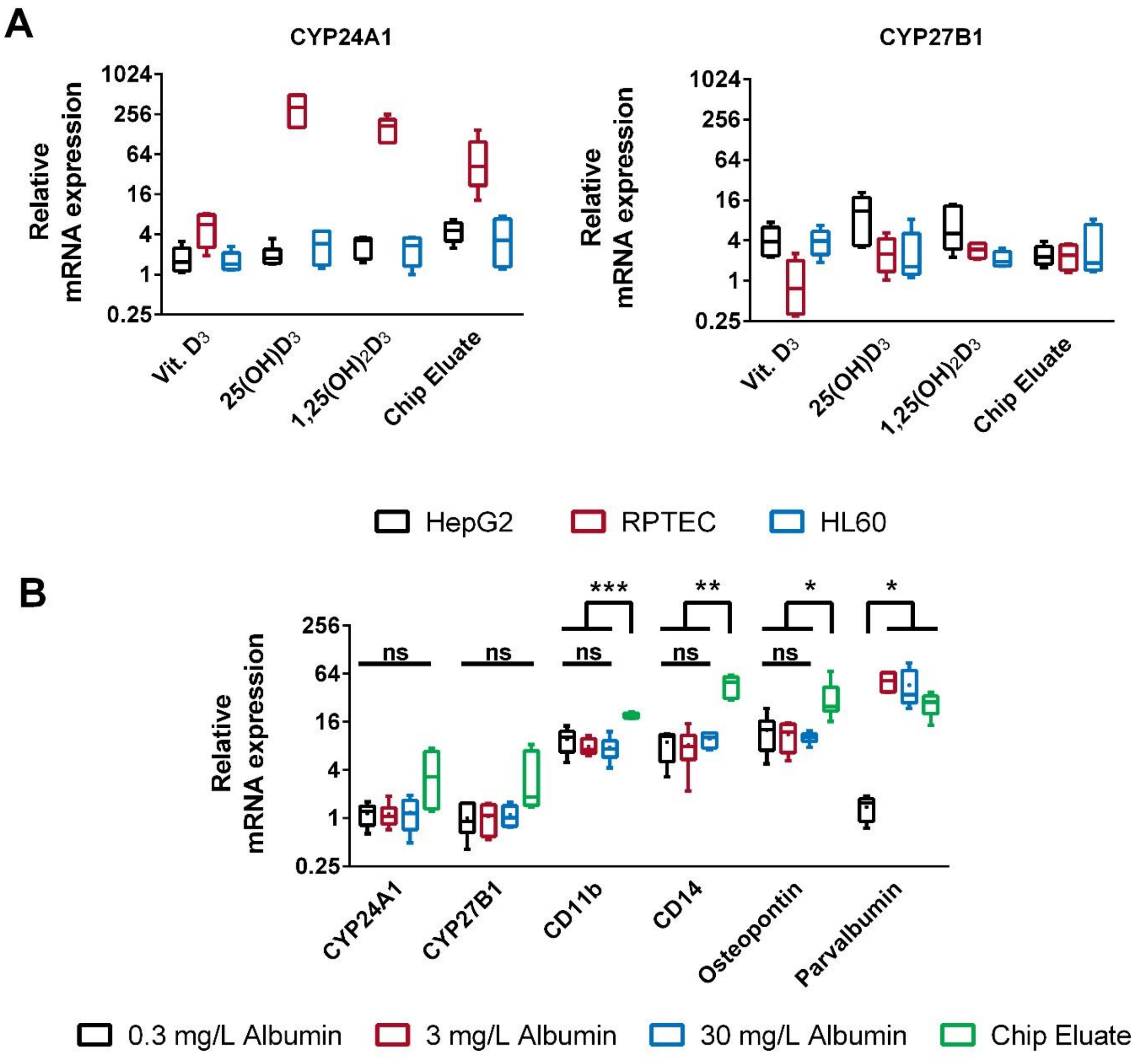

Supplementary Figure 2 ***A*** *Comparison of relative mRNA expression of CYP24A1 and CYP27B1 in HepG2, RPTEC and HL60 cells after treatment with Vit D3. 25(OH)D3 1,25(OH)2D3 and treatment with chip eluate using RT-qPCR.* ***B*** *Comparison of HL60 cells treated for 24 hours with 100nM 1,25(OH)2D3 and various concentrations of albumin compared to the chip eluate. Cells were treated for 24 hours for each condition. For all figures lines in the middle of the box plot represent the median, whereas the + sign represents the mean of more than 4 independent experiments. The statistical significance was calculated using an unpaired two-tailed Student's t-test, where p-values less than or equal to 0.05, 0.01, and 0.001, depicted as *, **, and ***, respectively*

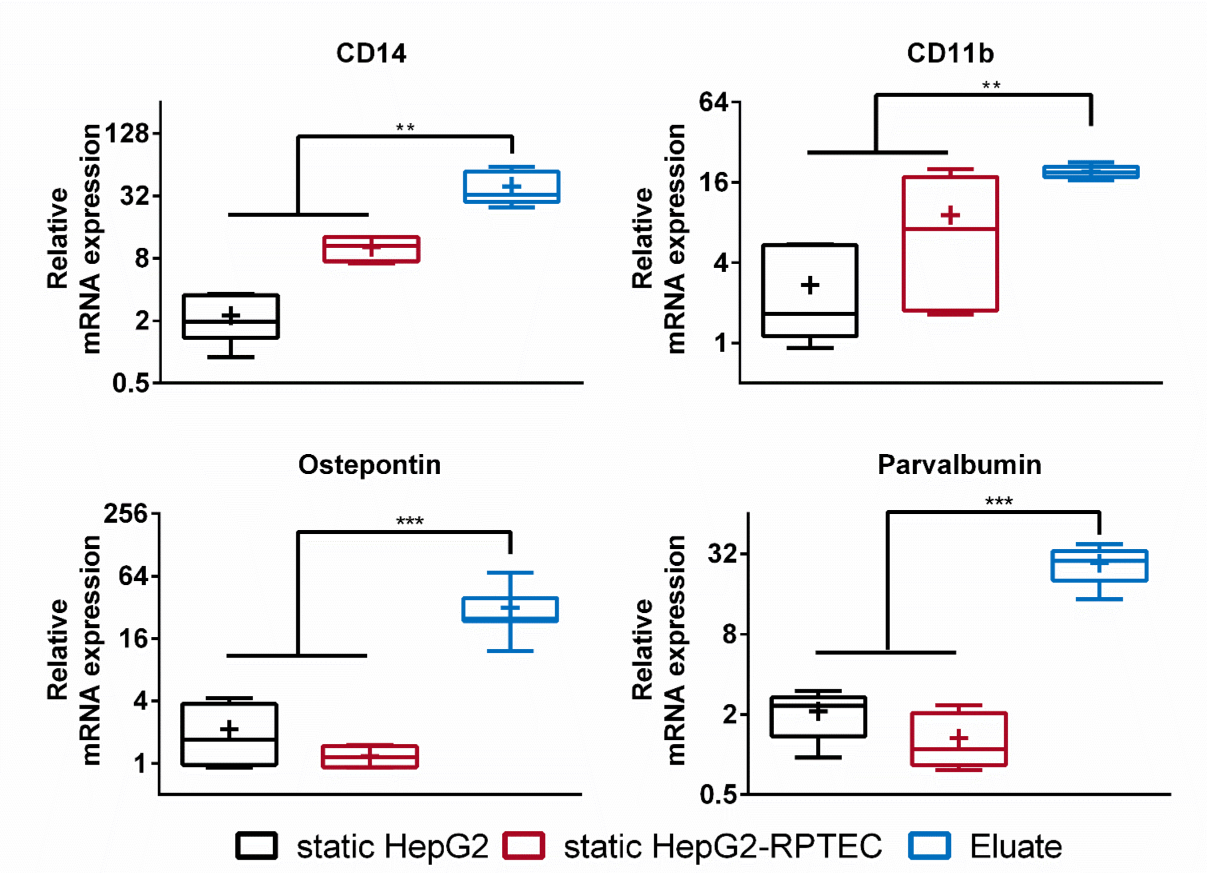

*Supplementary Figure 3 Effect of 20µM Vit D3 treatment in various experimental setups on the mRNA expression of multiple differentiation markers (CD14, CD11b, Osteopontin and Parvalbumin) in HL60 cells using RT-qPCR. Note that static HepG2 refers to the supernatant of HepG2 cells being treated with 20µM for 24 hours, static HepG2-RPTEC refers to the supernatant of HepG2 cells being treated with 20µM for 24 hours and RPTEC cells being treated with this supernatant for 24h and chip eluate refers to the eluate of a chip containing both HepG2 and RPTEC cells in subsequent chambers, which was fed with medium containing Vit. D3. For all figures lines in the middle of the box plot represent the median, whereas the + sign represents the mean of more than 4 independent experiments Error bars ± SD Statistical significance between the cells treated with the chip eluate and other treatments was calculated using an unpaired two-tailed Student's t-test, where p-values less than or equal to 0.05, 0.01, and 0.001, depicted as *, **, and ***, respectively.*

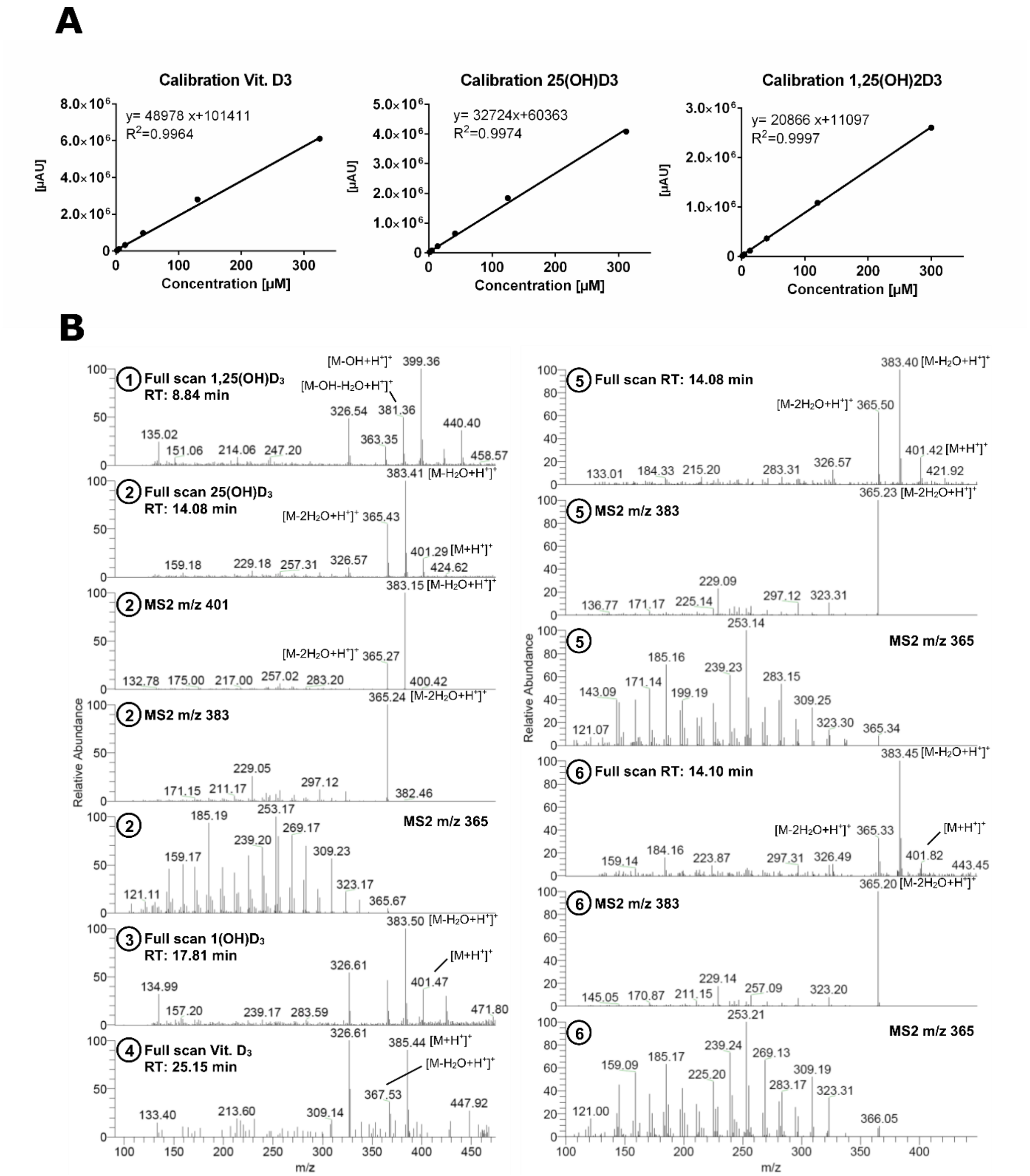

Supplementary Figure 4 ***A*** *Calibration curves for HPLC peaks of Vit. D3, 25(OH)D3, and 1,25(OH)2D3. Linear curve fit was used to calculated unknown concentration in the sample supernatants and eluates.* ***B*** *1-4: Full scans of all standards (compare Figure 5A) and fragmentation patterns of the molecule ion and the fragment ions by source of 25(OH)D_3 (2). ~~Lower half right~~ 5-6: Verification of the metabolite 25(OH)D_3 in samples V (figure 5)peak (5) and VI (figure 5) peak (6) by full scans and fragmentation patterns. RT: Retention time.*
